# Seltene Erkrankungen – die Rolle der Inneren Medizin

**DOI:** 10.1007/s00108-025-01892-7

**Published:** 2025-04-24

**Authors:** Tilman Sauerbruch, Stefan Aretz, Helge Hebestreit, Harald Kaemmerer, Lutz Nährlich, Britta Siegmund, Georg Ertl, Deutsche Gesellschaft für Innere Medizin (DGIM)

**Affiliations:** 1 Medizinische Klinik I, Universitätsklinikum Bonn, Venusberg-Campus 1, 53127 Bonn, Deutschland; 2 Institut für Humangenetik, Zentrum für erbliche Tumorerkrankungen, Biomedizinisches Zentrum, Universitätsklinikum Bonn AöR, Venusberg-Campus 1, Bonn, Deutschland; 3 Universitätsklinikum Würzburg, Würzburg, Deutschland; 4 Deutsches Herzzentrum München, Klinikum der Technischen Universität München, München, Deutschland; 5 Zentrum für Kinderheilkunde und Jugendmedizin, Abteilung Allgemeine Pädiatrie und Neonatologie, Funktionsbereich Pädiatrische Pneumologie und Allergologie, Universitätsklinikum Gießen-Marburg GmbH, Gießen, Deutschland; 6 Medizinische Klinik für Gastroenterologie, Infektiologie, Rheumatologie (einschl. Ernährungsmedizin), Charité – Universitätsmedizin Berlin, Campus Benjamin Franklin, Berlin, Deutschland; 7 Deutsches Zentrum für Herzinsuffizienz, Universitätsklinikum Würzburg, Würzburg, Deutschland

**Keywords:** Seltene Erkrankungen/Versorgungsstrukturen, Seltene Erkrankungen/Diagnostik, Referenznetzwerke, Orphan Drugs, Transition in die Erwachsenenmedizin, Rare diseases/health care structures, Rare diseases/diagnosis, Reference networks, Drugs, orphan, Transition to adult care

## Abstract

Seltene Erkrankungen, in der Europäischen Union definiert als Leiden, die weniger als 5 von 10.000 Einwohnern betreffen, werden häufig schon im Kindesalter manifest, spielen aber aufgrund der deutlich verbesserten Langzeitprognose und auch einer Reihe primär erst im Erwachsenenalter auftretender Erkrankungen in der Inneren Medizin eine zunehmende Rolle. Obwohl national und international bereits beachtenswerte Strukturen bestehen (Netzwerke, Register, Datenbanken, Selbsthilfegruppen), müssen die Aufmerksamkeit für diese Erkrankungen in der täglichen Routine und die Kenntnis der teilweise divergenten Versorgungsstrukturen verbessert werden. Für viele seltene Erkrankungen gibt es keine spezifischen Therapien, aber es werden – gerade in der Onkologie – zunehmend Medikamente entwickelt, die einem gesonderten Orphan-Drug-Status unterliegen.

## Abstract (en)

Rare diseases, defined in the European Union as conditions affecting fewer than five per 10,000 inhabitants, often manifest themselves in childhood, but are playing an increasingly important role in internal medicine due to the significantly improved long-term prognosis and a number of diseases that primarily occur in adulthood. Although noteworthy structures already exist nationally and internationally (networks, registers, databases, self-help groups), awareness of these diseases in daily routine and knowledge of the partly divergent care structures must be improved. There are no specific treatments for many of these diseases, but drugs are increasingly being developed—particularly in oncology—that are subject to special orphan drug status.

Kardiovaskuläre, metabolische oder maligne Erkrankungen – häufig dem modernen Lebensstil geschuldet – belasten weltweit die Gesundheitssysteme. Gleiches gilt für pandemisch auftretende Infektionen und neurodegenerative Leiden im fortgeschrittenen Alter [1]. Bei den seltenen Erkrankungen (SE) liegen Prävalenz und Inzidenz im Vergleich zu den Volkskrankheiten deutlich niedriger. Dennoch haben SE insgesamt eine Prävalenz von über 5 % in Europa. Diese verteilt sich allerdings auf sehr viele unterschiedliche Leiden, nach derzeitiger Schätzung 5000 bis 10.000 [2], einige davon mit weniger als 100 weltweit bekannten Fällen. Das bedeutet, dass sie in nicht darauf spezialisierten internistischen Praxen und Kliniken gelegentlich oder nie vorkommen oder nicht als solche erkannt werden. Da erkrankte Organe – unabhängig von der Ursache – ein begrenztes klinisches Spektrum aufweisen, werden SE leicht einer häufigen Ätiologie zugeordnet, obwohl die Pathophysiologie hinter den Symptomen eine ganz andere ist als angenommen. Das Risiko falscher oder unzureichender Therapien ist daher gerade bei SE hoch.

Die ständige Verbesserung von Diagnostik, Therapien und Prävention der SE hat die Lebenserwartung vieler Betroffener deutlich verlängert. Das verlangt bei frühmanifesten SE einen möglichst optimalen Übergang in die Erwachsenenmedizin (Transition). Hier sind unterschiedliche Fachrichtungen gefordert, besonders die Innere Medizin, da viele Diagnosen in den Bereich ihrer Schwerpunkte fallen. So sind beispielsweise 2 Mio. Europäer von seltenen Nierenerkrankungen betroffen [3]. Auch viele erbliche Tumorsyndrome, deren Prognose sich durch eine frühe klinische oder genetische Diagnose und präventive Interventionen deutlich gebessert hat, fallen in Gebiete der Inneren Medizin.

## Definition

Die Europäische Union (EU) definiert SE als lebensbedrohliche oder chronische Einschränkung der Gesundheit, die bei weniger als 5 Menschen pro 10.000 Einwohner auftreten [4]. Viele andere Länder setzen die Schwelle ebenfalls auf einen Wert von etwa 1:2000 [5]. China und Russland definieren SE durch eine Prävalenz unter 1:10.000, in der Türkei gilt als Schwelle eine Prävalenz unter 1:100.000. In Indien, dem bevölkerungsreichsten Land der Erde, existiert keine offizielle Definition [6]. Aufgrund dieser Unterschiede lassen sich SE weltweit epidemiologisch und statistisch nicht einheitlich erfassen. Basierend auf den Definitionen schätzt man, dass in der EU etwa 30 Mio. Menschen (7 % der Bevölkerung) betroffen sind und in den USA 20–30 Mio. [7], weltweit 300 Mio. Menschen [8], verteilt auf 5000–10.000 Erkrankungen. Für Deutschland wird häufig die Zahl von 4 Mio. Betroffenen angegeben, bei Annahme einer Prävalenz von 7 % wären es noch mehr.

## Diagnose

Die häufigeren SE und solche mit klassischen Leitsymptomen sind in gängigen Lehrbüchern oder digitalen Quellen abgehandelt. Aufgrund der Seltenheit ihres Vorkommens ist das Wissen zu den jeweiligen Entitäten aber häufig nicht präsent. Die Patienten stellen sich mit ihren Symptomen vor und werden dementsprechend behandelt. Hohes Patientenaufkommen, Zeitnot und die geringe A‑priori-Wahrscheinlichkeit begünstigen dann die Fehldeutung der Symptome. Bei rezidivierenden chronischen Beschwerden ist die Chance einer korrekten Diagnose höher, aber der Weg dahin leider oft lang.

In der Kinderheilkunde werden einige behandelbare, meist auf Stoffwechselstörungen beruhende SE durch Neugeborenenscreening schon im asymptomatischen Stadium diagnostiziert [9, 10]. Deren Häufigkeit liegt zwischen 1/3300 (zystische Fibrose) und 1/1.000.000 (Carnitinstoffwechseldefekte). Ein solcher Screeningansatz ist jedoch für die überwiegende Mehrzahl der SE bisher nicht möglich oder sinnvoll. Die Diagnostik erfolgt symptomorientiert. Lediglich die familiäre Hypercholesterinämie kann im Rahmen des Gesundheits-Check-ups in Deutschland ab dem 18. Lebensjahr durch einmalige Bestimmung des Lipidprofils (hohes Low-density-Lipoprotein[LDL]-Cholesterin) im asymptomatischen Stadium erfasst werden.

Es wird geschätzt, dass etwa 80 % der SE primär auf einer genetischen Ursache beruhen. Meist handelt es sich um monogene erbliche Erkrankungen [11]. Mithilfe der modernen Hochdurchsatzdiagnostik lässt sich schon heute bei über 50 % aller Patienten, die von einer SE betroffen sind, eine genetische Ursache identifizieren; man schätzt, dass dadurch etwa 4500 Erkrankungen abgedeckt sind. Dennoch bleibt das Problem bestehen, die klinischen Leitsymptome für eine genetische Untersuchung zu definieren bzw. diese richtig zu erkennen. Noch schwieriger wird es, wenn die Beziehung zwischen genetischer Prädisposition und Phänotyp variabel ist. Bei exogen ausgelösten SE (etwa bei Asbestbelastung und unklarem Erguss aufgrund eines Pleuramesothelioms) hilft nur die sorgfältige Anamnese. Auch vom Immunsystem induzierte Erkrankungen spielen, gerade im Erwachsenenalter, eine Rolle in der Differenzialdiagnose von SE. Eine auffallend komplexe Symptomatik kann auf einer Kombination aus verschiedenen Krankheitsbildern von SE und Nicht-SE (unter anderem psychischen Störungen) beruhen [12].

Wahrscheinlich werden auf künstlicher Intelligenz (KI) basierende Systeme, welche Muster aus Anamnese, klinischer Untersuchung und Laborparametern analysieren und eine Verdachtsdiagnose generieren, den Weg zu einer korrekten Diagnose in Zukunft beschleunigen [13–17]. Viele Zentren für SE [18] haben Lotsenstrukturen unter Einbeziehung unterschiedlicher Fachrichtungen und telemedizinischer Sprechstunden eingerichtet, um die Diagnosefindung bei SE – und damit auch die Therapie – zu verbessern [12].

### Bedeutung der Diagnose einer erblichen seltenen Erkrankung

Die sichere Diagnose einer erblichen Erkrankung und gegebenenfalls vorhandener molekularer Subtypen ist oft nur durch die genetische Keimbahndiagnostik möglich. Sie erlaubt eine präzisere Einschätzung des Krankheitsverlaufs, präventive Maßnahmen und immer spezifischer werdende Therapien. Selbst bei nicht behandelbaren Erkrankungen entlastet die klare Diagnose von Unsicherheiten und Schuldzuweisungen. Risikopersonen aus einer betroffenen Familie kann eine prädiktive genetische Testung angeboten werden, die dann alle Nichtanlageträger von der Krankheitsbedrohung befreit und gegebenenfalls teure medizinische Maßnahmen verhindert. Gleichzeitig können den Anlageträgern gezielte Vorsorgemaßnahmen und Therapien angeboten werden – am besten in ausgewiesenen interdisziplinären Zentren. Die erblichen Tumorsyndrome sind hierfür ein gutes Beispiel.

## Therapie

Die Therapie richtet sich im Idealfall nach evidenzbasierten Leitlinien. Dabei müssen Besonderheiten der Erkrankungen und Komorbiditäten berücksichtigt werden. Dies ist nicht immer der Fall, so kommt es beispielsweise zur falschen Indikationsstellung oder inadäquaten Dosierung bei angeborenen Herzerkrankungen. Leider gibt es derzeit nur für weniger als 10 % aller SE eine gezielte Behandlung.

Medikamente, die spezifisch der Behandlung von SE dienen, unterliegen in den USA seit 1983 dem Orphan Drug Act und in der EU seit 2000 einer analogen Verordnung [4]. Voraussetzung für die Erlangung des Orphan-Drug-Status ist die Erfüllung der Definition einer SE, die zu einem lebensbedrohlichen oder chronischen Leiden führt und für die bisher kein zufriedenstellendes Arzneimittel vorhanden bzw. zugelassen ist. Der Nachweis des Nutzens beruht – bei vertretbaren Nebenwirkungen – auf einer gesicherten Verbesserung der Prognose und/oder Lebensqualität (Committee for Orphan Medicinal Products; [19]). Alle Entwicklungen unterliegen definierten Ansprüchen hinsichtlich Qualität, Unbedenklichkeit und Wirksamkeit. Die Medikamente für SE haben einen Sonderstatus auf dem Weg zur Zulassung und 10 Jahre Marktexklusivität nach der Zulassung. Im Jahr 2024 waren 150 Orphan Drugs in der EU zugelassen. Bei immer detaillierterer Kenntnis der Pathogenese der Erkrankungen wird auch die medikamentöse Therapie spezifischer. Beispiele sind

- der Einsatz von „small interfering RNA“ (siRNA) zur gezielten Hemmung der Translation bestimmter Proteine (beispielsweise zur Reduktion des abnormen Proteins Transthyretin bei der Amyloidose [20]),
- gezielte Antikörpertherapien (Hemmung des Enzyms PCSK9 bei schwerer Hypercholesterinämie [21]),
- die Unterbrechung von Signalkaskaden durch Hemmung von Tyrosinkinasen (unter anderem mit „small molecules“ wie Imatinib bei gastrointestinalen Stromatumoren oder chronischer myeloischer Leukämie [22]),
- die gezielte Blockierung von Transportern (Bulevirtid zur Entry-Inhibition bei Hepatitis D [23]) und
- eine gezielte Modulation von Ionenkanälen (beispielsweise mit CFTR-Modulatoren bei Mukoviszidose [24]).

Wegen der geringen Zahl Betroffener und der häufig komplexen Therapien sollten die Patienten möglichst ausgewiesenen Zentren zugewiesen werden.

Patienten mit seltener Erkrankung sollten möglichst ausgewiesenen Zentren zugewiesen werden

Auch für Orphan Drugs gilt die Forderung nach einem robusten Vergleich mit dem bisherigen Therapiestandard, am besten in Form randomisierter, kontrollierter Studien, um rationale Behandlungs- und Erstattungsentscheidungen zu ermöglichen. In der Entwicklung von Pharmaka zur gezielten Behandlung von SE stehen derzeit Onkologika im Fokus der Forschung und Anwendung, obwohl weniger als 10 % der SE der Onkologie zuzuordnen sind [25]. Nach einer Analyse des Instituts für Qualität und Wirtschaftlichkeit im Gesundheitswesen (IQWiG) wurde in Standard-Nutzenbewertungen nach Auslaufen des Orphan-Drug-Status für etwa die Hälfte der früheren Orphan Drugs kein Zusatznutzen nachgewiesen, vorwiegend weil robuste Vergleiche mit einem Therapiestandard fehlen [26]. Vor dem Hintergrund dieser Ergebnisse sollte eine Reform der Orphan Drug Regulation diskutiert werden [27].

## Transition

Viele SE treten bereits bei Kindern auf [28]. Durch Fortschritte in der Diagnostik und Therapie hat sich ihre Prognose in den letzten Jahrzehnten deutlich verbessert. Viele Betroffene erreichen das Erwachsenenalter und müssen daher zu gegebener Zeit aus der Pädiatrie in den medizinischen Versorgungsbereich für Erwachsene überführt werden, eine Phase, die als Transition zusammengefasst wird. Dieser Zeitraum ist für den Krankheitsverlauf eine vulnerable Periode [29, 30]. Die körperliche und psychische Entwicklung (Pubertät), das Eltern-Kind-Verhältnis (Zulassen von und Bereitschaft zur Eigenverantwortlichkeit), der Verlust fester Bezugspersonen in der medizinischen Versorgung und fehlendes Fachpersonal in der Erwachsenenmedizin sind Probleme, die zu Verschlechterungen führen können [30].

Die Gesellschaft für Transitionsmedizin e. V. adressiert in Zusammenarbeit mit den erkrankungsbezogenen und sozial relevanten Fachgesellschaften den Bereich der Transition in einer S3-Leitlinie [31], geht aber nicht auf die spezifischen Aufgaben bei den einzelnen Krankheitskomplexen ein. So leben in Deutschland mehr als 500.000 Kinder und Erwachsene mit angeborenen Herzfehlern, von denen einige zu den SE gehören. Die Verläufe dieser Erkrankungen bedürfen – beispielsweise in Bezug auf die Dosierung von Medikamenten, die Führung von Narkosen und Besonderheiten in der Schwangerschaft – einer speziellen Fortbildung und ausreichender Erfahrung [30, 32, 33], die im Facharztbereich der Erwachsenenmedizin häufig nicht gegeben ist. Auch fehlt oft die Bereitschaft, sich auf diese komplexen Herausforderungen einzulassen – aus Gründen wie Zeitnot, fehlender spezifischer Erfahrung und Ausbildung oder unzureichender Vergütung. Manche jungen Patientinnen und Patienten haben die Tendenz, bei den ihnen langjährig vertrauten Ärzten bzw. Institutionen zu bleiben. Andere Jugendliche empfinden den Wechsel im Sinne einer Selbstbestimmung positiv. Insgesamt besteht jedoch die Gefahr, durch Verlassen adäquater Nachsorgestrukturen die Erkrankung zu vernachlässigen [34].^.^ Die Notwendigkeit einer Transition sollte daher schon ab dem 12.–14. Lebensjahr in gemeinsamen Gesprächen unter Einschluss der Eltern adressiert werden. So können notwendige Ansprechpartner für die Behandlung im Erwachsenenalter rechtzeitig ausgemacht werden.

Derzeit werden junge und ältere Erwachsene mit SE häufig weiter von den ihnen vertrauten Pädiatern betreut, was bezüglich ihrer Grunderkrankung meist gut funktioniert, aber zu Schwierigkeiten bei der Abrechnung erbrachter ärztlicher Leistungen führen kann. Schwierig wird es, wenn in der Pädiatrie nicht übliche Komorbiditäten auftreten. Spätestens dann muss eine Transition erfolgen, idealerweise in einer Kooperation zwischen Pädiatrie und Erwachsenenmedizin.

## Strukturen

Auf nationaler und internationaler Ebene wurde die Notwendigkeit erkannt,

- SE rechtzeitig zu diagnostizieren,
- sie standardisiert digital zu erfassen,
- eine adäquate Behandlung und gegebenenfalls Prävention zu garantieren,
- die Forschung zu unterstützen (auch im Sinne struktureller Voraussetzungen für gute klinische Studien) und
- Fortbildung zu SE anzubieten.

Einige notwendige Voraussetzungen hierfür sind schon heute erfüllt. Sie betreffen nicht nur die medizinische Versorgung, sondern auch die Grundlagenforschung und die angewandte Forschung.

Sowohl auf EU- [35] als auch auf nationaler Ebene, wie beispielsweise in Deutschland [36] oder Großbritannien und den USA [37], werden Probleme in Bezug auf SE adressiert, auch durch die Unterstützung von Netzwerken oder Registern.

### Netzwerke

Erfreulicherweise existiert eine ganze Reihe von Netzwerken. Einige Beispiele:

- Zentren für seltene Erkrankungen (ZSE) – Bündelung von Krankenversorgung, Forschung und Lehre, Förderung der Interaktion verschiedener Fachdisziplinen in Deutschland und Aufbau von Lotsenstrukturen zur Leitung von Betroffenen an Expertenzentren [38]
- Deutsches Netzwerk für Personalisierte Medizin (DNPM) – eine Kooperation von 26 Universitätskliniken/ZSE zur Verbesserung der personalisierten Behandlung [39]
- Allianz Chronischer Seltener Erkrankungen (ACHSE) – gefördert durch Krankenkassen, Bundesministerium für Gesundheit (BMG) und Industrie; Information von Betroffenen, Konzepte für Versorgung und Forschung [40]
- GenomDE – eine nationale Initiative zur Verbesserung der Versorgung von Patienten mit SE und Tumorerkrankungen durch Implementierung der Genomsequenzierung in die Regelversorgung mit dem Modellvorhaben Genomsequenzierung nach § 64e Sozialgesetzbuch (SGB) V [41]
- Europäische Referenznetzwerke (ERN) – gefördert von der EU; Verbesserung der Gesundheitsversorgung bei SE in spezialisierten europäischen Krankenhäusern durch digitalen Wissenstransfer, Erstellung von Leitlinien, Öffentlichkeitsarbeit und individuelle Fallberatungen [35]
- Konsortien für familiäre Krebserkrankungen und erbliche Tumorsyndrome [42]
- Nationales Aktionsbündnis für Menschen mit Seltenen Erkrankungen (NAMSE) – Steuerung von Arbeitsgruppen im Bereich seltener Erkrankungen im Sinne von Patientinnen und Patienten mit seltenen Erkrankungen [43]

Darüber hinaus gibt es weitere deutsche Referenznetzwerke (DRN) für einige SE wie Autoinflammation, Lungenerkrankungen oder orofaziale Fehlbildungen. Sie basieren aktuell noch auf Empfehlungen der Arbeitsgemeinschaft der Zentren für Seltene Erkrankungen und auf Eigeninitiative. Im Rahmen der europäischen Joint Action JARDIN zur Integration der ERN in die nationalen Gesundheitssysteme werden zurzeit Empfehlungen zur Struktur und Funktionsweise nationaler Referenznetzwerke entwickelt. Einige Standorte in Deutschland sind auch Teil der ERN.

### Register

Die Erstellung möglichst grenzüberschreitender Register ist für SE eine wichtige Maßnahme. Sie erlauben es, unterschiedliche Phänotypen und Genotypen, Genotyp-Phänotyp-Beziehungen, die Effizienz von Therapien oder auch natürliche Krankheitsverläufe einschließlich prognostischer Faktoren zu erfassen [44]. Gut strukturierte Register können zudem für die Evaluation der Effekte neuer Therapien herangezogen werden, idealerweise auch zur Rekrutierung für randomisierte Studien [45]. Beispiele hierfür sind das Register für mitochondriale Erkrankungen [46], das Register für Mukoviszidose [47] oder das PATHFINDER-CHD-Register für herzinsuffiziente Erwachsene mit angeborenen Herzfehlern [48]. Die Einführung der elektronischen Patientenakte sollte es in Zukunft ermöglichen, Daten, die im klinischen Alltag anfallen, wissenschaftlich auszuwerten. Dies könnte für die SE-Forschung bedeutsam sein, vorausgesetzt die Qualität der Daten ist ausreichend und die datenschutzrechtlichen Hürden sind nicht zu hoch.

### Informationsstrukturen

Viele der oben genannten Netzwerke koordinieren bzw. formulieren Informationen für SE. Der Atlas für SE (SE-ATLAS) sammelt das Angebot von Zentren, die sich mit unterschiedlichen SE befassen [18]. Die gut recherchierten und kritisch geprüften Gesundheitsinformationen des IQWiG haben das Ziel, Informationen zu Krankheitsgruppen zu erstellen, von denen mindestens 1 % (Prävalenz oder Inzidenz) der Bevölkerung betroffen ist. Somit sind SE hier nur indirekt erfasst. Eine Reihe von Hinweisen findet sich auch auf der EU-geförderten Plattform Orphanet [49]*. *Umfassende, strukturierte und regelmäßig aktualisierte Informationen zu einer Vielzahl monogener erblicher Erkrankungen finden sich bei GeneReviews [50].

### Datenbanken und digitale Erfassung

Die Erfassung von SE erfolgt in unterschiedlichen Systemen, die an klinisch-phänotypischen, pathophysiologischen oder genetischen Parametern bzw. auch einer Kombination dieser Kriterien orientiert sind. Die Codierung der Internationalen statistischen Klassifikation der Krankheiten und verwandter Gesundheitsprobleme (ICD) erfasst eine Reihe von SE, aber einige auch nicht oder zu unspezifisch [51]. Online Mendelian Inheritance in Man (OMIM) sammelt Informationen zu monogenen Erkrankungen, die häufig zu den SE gehören [52]. Human Phenotype Ontology (HPO) erfasst und ordnet klinische Merkmale [53].

ORPHAcodes ist das international geltende Klassifizierungssystem für die Diagnosen von SE [54]. Die Alpha-ID-SE-Codierung verknüpft in Deutschland die ICD-10-GM- und ORPHA-Codes. Eine spezifische Codierung der SE ist damit möglich und für stationäre Fälle seit 2023 in Deutschland vorgeschrieben. Damit könnte auf längere Sicht ein genaueres Bild über Prävalenz und Verteilung von SE hierzulande entstehen.

## Probleme

- Die Definition von SE führt zu regional unterschiedlichen Diagnosen und auch einer unterschiedlichen Prävalenz von SE. Das betrifft beispielsweise Infektionserkrankungen. So wurden 2023 in Deutschland etwa 4500 Tuberkulosefälle registriert [55]. Das entspricht einer jährlichen Inzidenz von 5/100.000. In Europa erfüllt die Tuberkulose somit die Definition einer SE [49], während 2021 die Inzidenz in Simbabwe 190/100.000 betrug [56]. Das Phänomen möglicher regionaler Unterschiede wird bisher unzureichend adressiert.
- Derzeit ist schlecht erforscht, inwieweit der Klimawandel das Spektrum der SE in verschiedenen Regionen beeinflusst.
- Die Grenzen zu SE verschieben sich mit der zunehmenden Personalisierung von Diagnostik und Therapie. Vorwiegend auf der Sequenzierung von Tumorgewebe beruhende unterschiedliche molekulare „pathways“ machen Subtypen häufiger Krebsformen formal zu SE. Nach den Regularien der Europäischen Arzneimittel-Agentur (EMA) erhält eine so individualisierte Therapie allerdings nicht den Orphan-Drug-Status.
- Für viele Beteiligte ist es schwierig, die verschiedenen Netzwerke, Zentren, Register oder Taxonomien der Diagnosen zu verstehen. Auch dadurch kann die Diagnosefindung zu lange dauern [57]. Es sollten daher Überschneidungen vermieden werden. Um den Betroffenen diesbezüglich Hilfe anzubieten, wurde den Typ-A-Zentren in Deutschland eine Lotsenfunktion zugesprochen. Inwieweit die aktuellen Regelungen jedoch ausreichen, ist noch nicht abschätzbar.
- Bei der Befragung der Schwerpunkte der Inneren Medizin wurde mehrfach gefordert, hausärztlich tätige Ärztinnen und Ärzte gezielter auf SE hinzuweisen. Aber selbst bei besserer Aufklärung ist das Problem der Zeitknappheit nicht gelöst.
- Bei der Entwicklung neuer Medikamente existiert eine starke Fokussierung auf onkologische Erkrankungen. Auch bei nichtonkologischen SE sollte die Entwicklung spezifischer Therapien intensiviert werden, immer unter der Prämisse möglichst hoher Evidenzkriterien.

## Rolle der Deutschen Gesellschaft für Innere Medizin

Da SE des Erwachsenenalters häufig in den Bereich der Inneren Medizin fallen, hat sich die Deutsche Gesellschaft für Innere Medizin (DGIM) mit Vertretern ihrer Schwerpunkte, der Deutschen Gesellschaft für Kinder- und Jugendmedizin, der Deutschen Gesellschaft für Neurologie und wichtigen Akteuren auf dem Gebiet der SE zusammengefunden, um eine gemeinsame Position für zukünftige Aktivitäten zu finden. Die Wahrnehmung in Bezug auf SE sollte geschärft werden, insbesondere auch durch eine anhaltende und lebendige Interaktion zwischen spezialisierten Zentren und den hausärztlich tätigen Kolleginnen und Kollegen.

Die DGIM strebt folgende Ziele an:

- Ausbau der jetzt schon vorhandenen Arbeitsgruppe „Transition“ mit Ausweitung auf das Gesamtthema SE und Humangenetik in der Inneren Medizin
- Gezielte Hinweise auf die bereits vorhandenen Strukturen und Versorgungspfade, insbesondere auf vorhandene Netzwerke
- Beteiligung an der Erfassung von SE in den verschiedenen Schwerpunkten und Definition von Erkrankungen im Bereich der Erwachsenenmedizin als neue SE
- Enge Zusammenarbeit mit den Bereichen der humangenetischen Diagnostik und Patientenversorgung, insbesondere mit der Deutschen Gesellschaft für Humangenetik, sowie mit den etablierten Netzwerken im Bereich SE
- Förderung der Aufmerksamkeit für und Weiterbildung zu SE in den jeweiligen Schwerpunkten, auch hinsichtlich des Dialogs zwischen den Schwerpunkten (Internistenkongress, regelmäßige digitale Fortbildungen, Unterstützung von regelmäßigen Publikationen unter der Rubrik „Seltene Erkrankungen“ im Organ der DGIM *Die Innere Medizin*)
- Unterstützung einer lebensbegleitenden, sektorübergreifenden Betreuung von Patienten mit SE, hier besonders der Interaktion hausärztlich tätiger Internistinnen und Internisten mit den jeweiligen Schwerpunkten, auch unter Einbeziehung der ambulanten spezialfachärztlichen Versorgung (ASV), der medizinischen Versorgungszentren (MVZ) und der SE-Zentren
- Sondierung der Rolle und Unterstützung der Entwicklung von KI zur Beschleunigung der Diagnosefindung bei SE und auch in der Weiterbildung über SE (Simulation von Krankheitsbildern)
- Unterstützung telemedizinischer Strukturen in Gebieten mit geringer Bevölkerungsdichte zur Diagnostik und Versorgung von Patienten mit SE
- Gezielte und regelmäßige Informations- und Fortbildungsangebote für hausärztlich tätige Internisten, die eine Lotsenfunktion für Patienten mit SE haben. Insbesondere zur Steigerung der Wahrnehmung und zur Vermittlung von digitalen Konferenzen zusammen mit den entsprechenden Zentren für SE
- Unterstützung der Forschung auf dem Gebiet SE

## Fazit für die Praxis

- Das Risiko falscher Diagnosen und unzureichender Therapien ist bei seltenen Erkrankungen (SE) hoch.
- Orphan Drugs haben einen Sonderstatus auf dem Weg zur Zulassung und 10 Jahre Marktexklusivität nach der Zulassung. Auch für Orphan Drugs gilt die Forderung nach einem robusten Vergleich mit dem bisherigen Therapiestandard.
- Viele Kinder, die von SE betroffen sind, erreichen das Erwachsenenalter und müssen daher aus der Pädiatrie in die Erwachsenenmedizin überführt werden. Die Notwendigkeit einer Transition sollte früh in gemeinsamen Gesprächen unter Einschluss der Eltern adressiert werden.
- National und international wurde die Notwendigkeit erkannt, SE rechtzeitig zu diagnostizieren, sie standardisiert digital zu erfassen, eine adäquate Behandlung zu garantieren, die Forschung zu unterstützen und Fortbildung zu SE anzubieten.
- Probleme in Bezug auf SE werden auf europäischer und nationaler Ebene adressiert, auch durch die Unterstützung von Netzwerken oder Registern.
- Die Wahrnehmung in Bezug auf SE sollte geschärft werden, auch durch eine anhaltende und lebendige Interaktion zwischen spezialisierten Zentren und den hausärztlich tätigen Kollegen.
